# Longitudinal serological assessment of type VI collagen turnover is related to progression in a real-world cohort of idiopathic pulmonary fibrosis

**DOI:** 10.1186/s12890-021-01684-3

**Published:** 2021-11-23

**Authors:** Henrik Jessen, Nils Hoyer, Thomas S. Prior, Peder Frederiksen, Sarah R. Rønnow, Morten A. Karsdal, Diana J. Leeming, Elisabeth Bendstrup, Jannie M. B. Sand, Saher B. Shaker

**Affiliations:** 1 Biomarkers and Research, Nordic Bioscience, Herlev Hovedgade 205-207, 2730 Herlev, Denmark; 2 Department of Respiratory Medicine, Herlev and Gentofte University Hospital, Copenhagen, Denmark; 3 Department of Respiratory Disease and Allergy, Center for Rare Lung Diseases, Aarhus University Hospital, Aarhus, Denmark

**Keywords:** Type VI collagen, Biomarkers, Cohort study, Extracellular matrix, Idiopathic pulmonary fibrosis

## Abstract

**Background:**

Remodeling of the extracellular matrix (ECM) is a central mechanism in the progression of idiopathic pulmonary fibrosis (IPF), and remodeling of type VI collagen has been suggested to be associated with disease progression. Biomarkers that reflect and predict the progression of IPF would provide valuable information for clinicians when treating IPF patients.

**Methods:**

Two serological biomarkers reflecting formation (PRO-C6) and degradation (C6M) of type VI collagen were evaluated in a real-world cohort of 178 newly diagnoses IPF patients. All patients were treatment naïve at the baseline visit. Blood samples and clinical data were collected from baseline, six months, and 12 months visit. The biomarkers were measured by competitive ELISA using monoclonal antibodies.

**Results:**

Patients with progressive disease had higher (*P* = 0.0099) serum levels of PRO-C6 compared to those with stable disease over 12 months with an average difference across all timepoints of 12% (95% CI 3–22), whereas C6M levels tended (*P* = 0.061) to be higher in patients with progressive disease compared with stable patients over 12 months with an average difference across all timepoints of 12% (95% CI − 0.005–27). Patients who did not receive antifibrotic medicine had a greater increase of C6M (*P* = 0.043) compared to treated patients from baseline over 12 months with an average difference across all timepoints of 12% (95% CI − 0.07–47). There were no differences in biomarker levels between patients receiving pirfenidone or nintedanib.

**Conclusions:**

Type VI collagen formation was related to progressive disease in patients with IPF in a real-world cohort and antifibrotic therapy seemed to affect the degradation of type VI collagen. Type VI collagen formation and degradation products might be potential biomarkers for disease progression in IPF.

**Supplementary Information:**

The online version contains supplementary material available at 10.1186/s12890-021-01684-3.

## Introduction

Idiopathic pulmonary fibrosis (IPF) is a fatal lung disease characterized by progressive worsening of lung function and a higher mortality rate than most cancers [1, 2]. Although IPF is progressive in nature, predicting the course of the disease in an individual patient is difficult [3, 4]. The antifibrotic therapies nintedanib and pirfenidone slow down the rate of decline in forced vital capacity (FVC) [5, 6]. Nintedanib is a tyrosine kinase inhibitor acting on platelet-derived growth factor, vascular endothelial growth factor, and fibroblast growth factor receptor, while pirfenidone probably acts by inhibiting transforming growth factor beta and tumor necrosis factor alpha [7, 8]. The American Food and Drug Administration (FDA) stated that there is a great need for finding novel tools to support the effects of the approved drugs [9]. Furthermore, the American Thoracic Society (ATS) recommends that future studies in pulmonary fibrosis should focus on finding new appropriate clinical endpoints, e.g. reflecting the degree of fibrosis and tissue turnover, to support functional assessments using FVC [10].

A central mechanism in IPF pathogenesis is the accumulation of extracellular matrix (ECM) proteins forming fibrotic tissue in the lungs and an imbalance in tissue remodeling and repair. Collagens are the main components of the ECM and their synthesis and degradation is strictly regulated, playing a critical role in the structure and function of healthy lungs [11–13]. Type VI collagen is a beaded filament collagen and is located in the interface between the basement membrane and interstitial matrix where it helps cell attachment to the surrounding matrix [14, 15]. Excess levels of both protein and mRNA of type VI collagen have been reported in patients with pulmonary fibrosis compared to healthy control [16], thus type VI collagen could play a central role in the pathogenesis of IPF.

When type VI collagen is secreted to the ECM, a specific fragment of the carboxyterminal C5 domain from the α3 chain of type VI collagen is cleaved off [17]. This fragment is named endotrophin and has been revealed as a potent driver of fibrosis in adipose tissue [18, 19]. Aligned with this, in the PROFILE (Prospective Observation of Fibrosis in the Lung Clinical Endpoints) cohort it was shown that PRO-C6, a serological neoepitope biomarker of endotrophin that also reflects type VI collagen formation, was elevated in patients with progressive IPF compared to patients with stable IPF [20]. Collectively, these data indicate that type VI collagen, specifically endotrophin, may play a fundamental role in fibrosis development and progression in IPF. Another biomarker of type VI collagen, C6M, is a neoepitope biomarker that reflects its degradation mediated by matrix metalloproteinase (MMP)-2 and -9 [21]. Serum levels of C6M have been found elevated in patients with progressive disease compared to patients with stable IPF [20, 22].

Since type VI collagen turnover has been associated with disease progression in patients with more advanced disease than those usually included in clinical trials, the aim of the current study was to evaluate (1) longitudinal serological assessments of type VI collagen turnover in stable and progressive IPF patients during a one-year period, and (2) whether antifibrotic therapy has an impact on type VI collagen turnover.

## Materials and methods

### Patient cohort

The Pulmonary Fibrosis Biomarker (PFBIO) cohort is an ongoing prospective cohort recruiting incident patients with IPF from two large interstitial lung disease (ILD) centers in Denmark. The PFBIO cohort has been described in details elsewhere [23]. The current study includes the first 178 patients from PFBIO with data from baseline, six and 12 month visits. An overview of the participants at each visit including the type of analyses can be found in Additional file 1: Fig. S1. Patients were enrolled if they had a diagnosis of IPF according to the 2011 ATS/ERS/JRS/ALAT guidelines [24]. Patients were included within two months from the day of their diagnosis. All patients were treatment naïve at the baseline visit. Two approved antifibrotic drugs were available, nintedanib and pirfenidone; the choice between them was up to the treating clinician and mainly based on the presence of potential absolute or relative contraindications and discussion of the side effect profile with the patients. During follow-up, patients were considered treated, if they had received at least one dose of nintedanib or pirfenidone and patients that did not receive antifibrotic treatment were considered untreated. Several measures of disease severity, including forced vital capacity (FVC), diffusion capacity for carbon monoxide (DLCO) and six-minute walk test (6MWT), were collected from the patients’ electronic records at baseline, six and 12 months. All patients provided written informed consent and the cohort was approved by the Regional Ethics committee (H-16001790) and the Danish Data Protection Agency (HGH-2016-017). The PFBIO study was registered at http://clinicaltrials.gov (NCT02772549) on April 29, 2016.

### Serum sampling and quantification of type VI collagen biomarkers

Serum samples were collected at baseline, six, and 12 months. Study specific operating procedures were used to ensure that there was as little variation as possible. Briefly, blood was collected into serum tubes and left undisturbed at room temperature to allow the blood to clot for 30–60 min. Serum was separated by centrifugation at 1300 × *g* for 10 min at 4 °C and aliquoted before storage at − 80 °C within 2 h from sample collection. Serum samples were analyzed by specific competitive ELISAs utilizing monoclonal antibodies for the C-terminal of the released C5 domain of type VI collagen α3 chain, also reflects type VI collagen formation (PRO-C6, cat. no. 4000) and for a neoepitope generated by MMP-2 and -9 degradation of type VI collagen (C6M, cat. no. 1500), as previously described (Nordic Bioscience, Herlev, Denmark) [21, 25]. All samples were run in duplicate and with an acceptance of CV% lower than 15%. Samples with a CV% higher than 15 were rerun.

### Statistical analyses

Descriptive data are presented as frequency tables or mean (SD) as appropriate for the data. A chi-squared test was used to detect differences in frequency data and an ANOVA test was used to detect mean differences between treatment groups. A t-test was used to detect mean differences between stable and progressive IPF patients. Disease progression was defined as an absolute decline in the percentage of predicted FVC ≥ 5% points and/or an absolute decline in the percentage of predicted DLCO  ≥ 10% points and/or all-cause mortality within 12 months. A linear mixed effects model was used to investigate associations between longitudinal biomarker measurements and disease progression. The model included log transformed biomarker levels as a dependent variable and progression status, visit, interaction between progression status and visit, age and sex as covariates. The model used a compound symmetry covariance structure. Missing lung function data were not imputed. The model implicitly imputes missing biomarker measurements and provides valid inference assuming they are missing at random. Since the missing at random assumption may be inappropriate if the biomarker measurements are missing due to death, we performed sensitivity analyses by restricting the study population to survivors only. Adjusted biomarker geometric means and 95% confidence interval (CI) by progression status at baseline, six months and 12 months and *p*-values of the test of no difference in means between stable and progressive patients were reported. A similar model was used to test for differences in biomarker levels between treatments groups (nintedanib, pirfenidone and no treatment). Furthermore, we analyzed differences in percentage change from baseline between treated and untreated groups. A similar model from the treatment analyses was employed and we included changes in biomarker levels as a dependent variable and treatment status (treated or untreated), visit, age, gender and PRO-C6 or C6M baseline levels as covariates.

Significance was accepted at a *p*-value of < 0.05. All statistical analyses were conducted with R (version 3.5.1) [26].

## Results

### Baseline characteristics

A total of 178 patients diagnosed with IPF were included in the current analyses and their baseline characteristics are summarized in Table 1 and Additional file 1: Table S1. At enrollment, median age were 73 years, 76% of patients were men, median FVC was 89.5% predicted and median DLCO was 52.8% predicted. The majority of patients (79.7%) were treated with nintedanib or pirfenidone after enrollment and at baseline there was a difference in smoking status between treatments (nintedanib, pirfenidone and no treatment). Patients without treatment were older and had shorter 6MWT distance (Table 1). Half of patients (47.2%) had progressive disease as defined 12 months after diagnosis. There was no difference in the number of patients with progressive disease between the different treatment groups (Table 1 and Additional file 1: Table S1).

**Table 1:** Baseline characteristics

Parameters	All patients n = 178	Nintedanib n = 62	Pirfenidone n = 80	No treatment n = 36	*p*-value
Age, mean (SD)	73.80 (7.53)	72.16 (7.15)	73.66 (6.87)	76.92 (8.74)	0.0097
Men, n (%)	136 (76.4%)	47 (75.8%)	62 (77.5%)	27 (75.0%)	0.95
BMI, mean (SD)	27.31 (4.51)	27.62 (4.62)	27.57 (4.16)	26.16 (5.02)	0.224
FVC (L), mean (SD)	3.04 (0.86)	3.05 (0.91)	3.08 (0.83)	2.94 (0.88)	0.7
FVC (% pred), mean (SD)	89.51 (19.52)	88.48 (19.57)	90.09 (18.71)	90.00 (21.65)	0.878
DLCO (% pred), mean (SD)	52.85 (13.25)	53.81 (12.68)	52.63 (13.13)	51.58 (14.8)	0.725
Change in FVC% pred. from baseline to 12 months, mean (SD)	0.13 (10.16)	0.20 (9.92)	0.06 (10.44)	0.15 (10.41)	0.997
Change in DLCO% pred. from baseline to 12 months, mean (SD)	− 5.03 (7.98)	− 4.70 (6.83)	− 5.37 (9.10)	− 4.85 (7.34)	0.9
6MWT (meters), mean (SD)	442.61 (105.58)	475.92 (95.2)	436.09 (106.85)	390.97 (101.61)	0.0009
*Smoking status, n (%) *					
Never	46 (25.8%)	10 (16.1%)	25 (31.2%)	11 (30.6%)	0.039
Active	11 (6.2%)	1 (1.6%)	6 (7.5%)	4 (11.1%)	
Former	121 (68.0%)	51 (82.3%)	49 (61.2%)	21 (58.3%)	
*GAP index, n (%) *					
I	88 (49.7%)	34 (54.8%)	36 (45.0%)	18 (51.4%)	0.08
II	82 (46.3%)	27 (43.5%)	42 (52.5%)	13 (37.1%)	
III	7 (4.0%)	1 (1.6%)	2 (2.5%)	4 (11.4%)	
Progression at 12 months, n (%)	84 (47.2%)	27 (43.5%)	38 (47.5%)	19 (52.8%)	0.67

SD standard deviation, BMI Body Mass Index, FVC forced vital capacity, DLCO diffusion capacity for carbon monoxide, 6MWT six-minute walk test, GAP index Gender-Age-Physiology index, Disease progression was defined as ≥ 5% decline in FVC and/or ≥ 10% decline in DLCO or all-cause mortality within 12 months.

### Longitudinal biomarker levels were elevated in progressive IPF

**Fig. 1:**
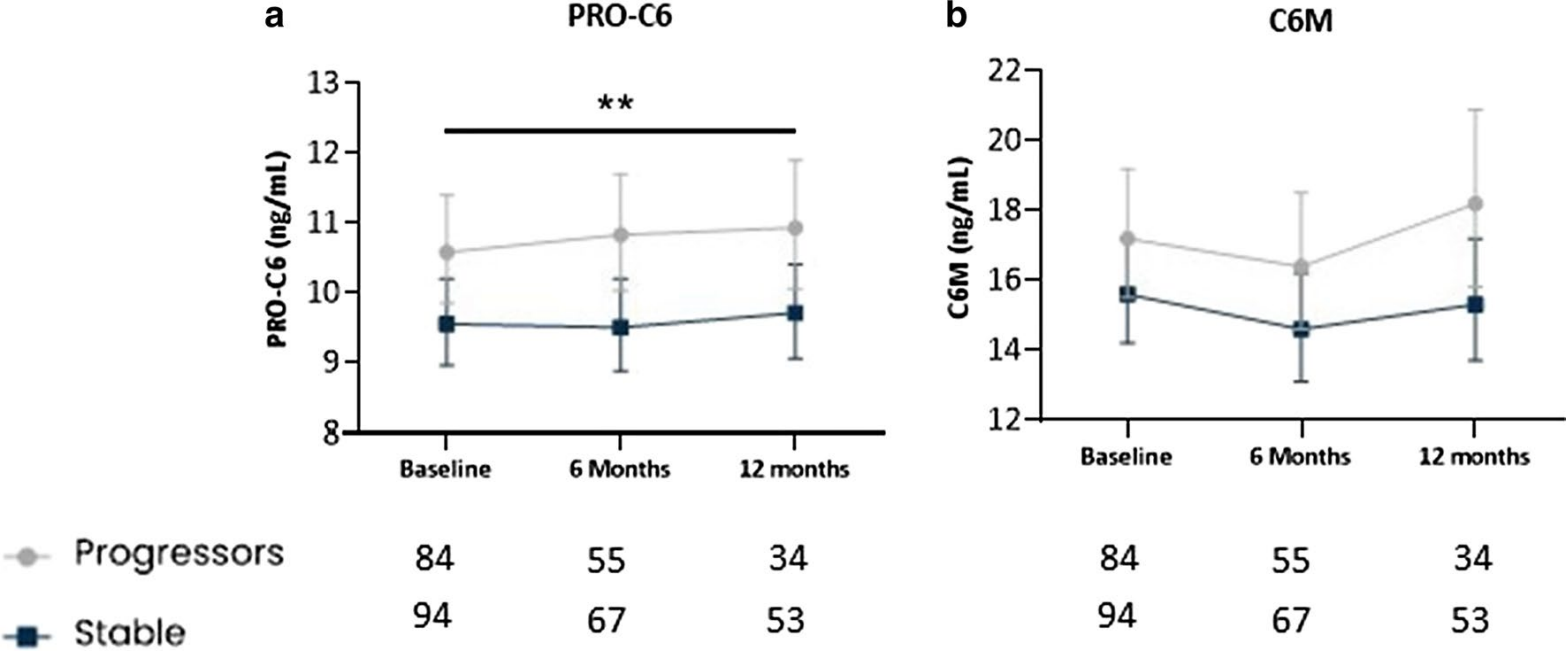
Longitudinal biomarker levels are elevated in progressive IPF patients. Serum levels of PRO-C6 (**A**) and C6M (**B**) are shown at baseline, six months and 12 months for stable (dark blue) and progressive (grey) patients with IPF. Disease progression was defined as ≥ 5% decline in FVC and/or ≥ 10% decline in DLCO or all-cause mortality within 12 months. Data are presented as geometric mean with 95% CI (error bars) adjusted for age and sex. The number of evaluable samples available for analysis at each time point is provided below the graphs. The interaction between timepoint and progression status was not significant for PRO-C6 (*P* = 0.66) and for C6M (*P* = 0.67). A significant average contrast across all timepoints between stable and progressors for PRO-C6 is shown as ** (*P* < 0.01)

Longitudinal biomarker levels were compared between IPF patients with stable disease and patients with progressive disease. Patients with progressive disease had significantly higher (*P* = 0.0099) serum levels of PRO-C6 compared to those with stable disease over 12 months with an average difference across all timepoints of 12% (95% CI 3–22) (Fig. 1A). Serum levels of C6M tended to be higher (*P* = 0.061) in patients with progressive disease compared with stable patients over 12 months with an average difference across all timepoints of 12% (95% CI − 0.005–27) (Fig. 1B). Sensitivity analyses excluding patients who died showed that patients with progressive disease had significantly higher (*P* = 0.035) serum levels of PRO-C6 compared to those with stable disease over 12 months with an average difference across all timepoints of 10% (95% CI 1–21) (Additional file 1: Fig. S2A). Furthermore, C6M tended to be higher (*P* =  0.09) in patients with progressive disease compared with stable patients over 12 months with an average difference across all timepoints of 12% (95% CI − 9–27) (Additional file 1: Fig. S2B).

### Longitudinal biomarker levels in treated and untreated IPF patients

**Fig. 2:**
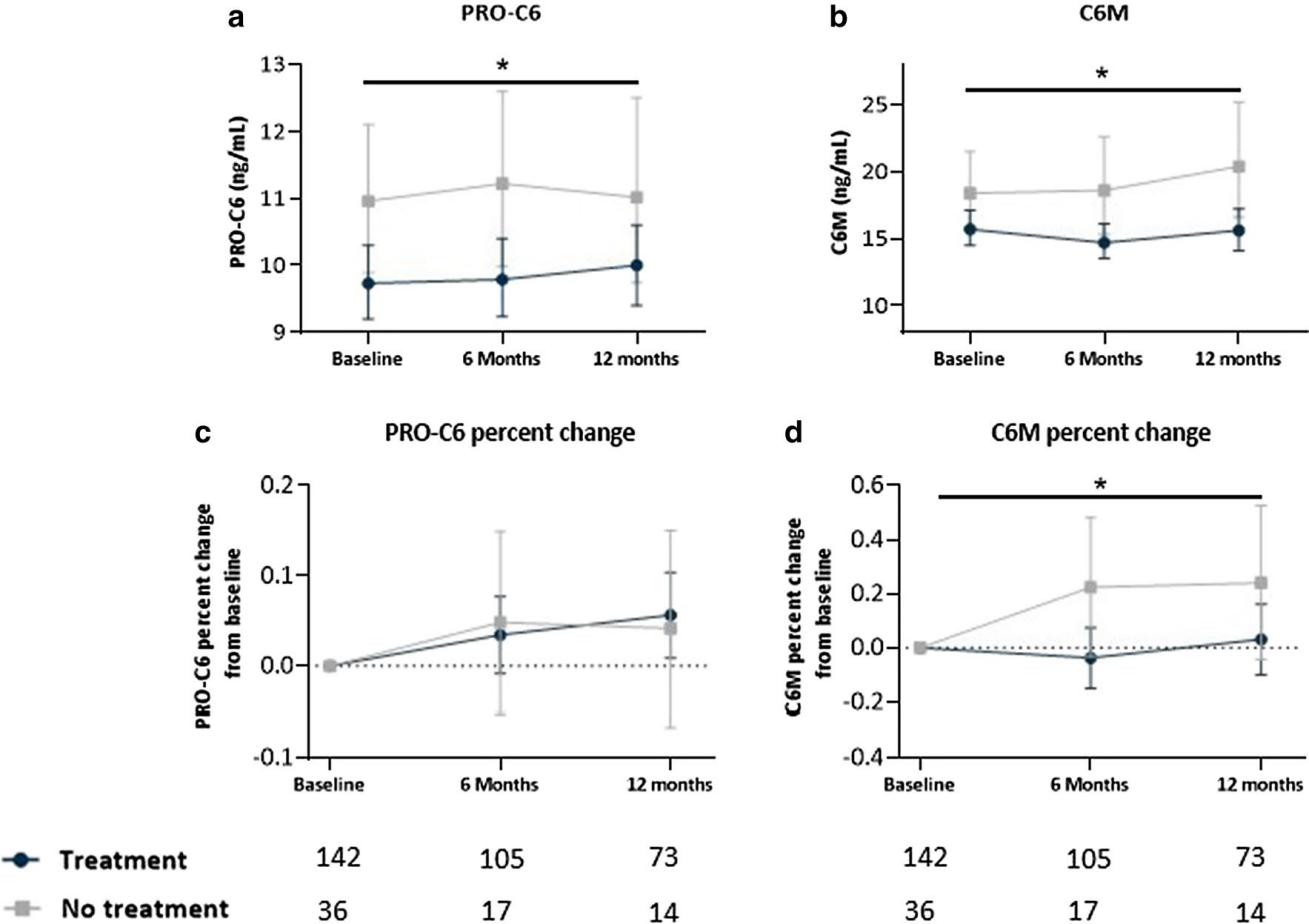
Longitudinal levels of type VI collagen biomarkers in treated and untreated IPF patients. Serum levels of PRO-C6 (**A**) and C6M (**B**) are shown at baseline, six months and 12 months for treated (nintedanib/pirfenidone) (dark blue) and for non-treated (grey); percent change from baseline in PRO-C6 (**C**) and, C6M (**D**) at six months and 12 months for treated (dark blue) and non-treated (grey) patients with IPF. Data are presented as geometric mean with 95% CI (error bars) adjusted for age and sex (**C** and **D** were also adjusted for PRO-6 or C6M baseline levels). The number of evaluable samples available for analysis at each time point is provided below the graphs. The interaction between timepoint and treatment was not significant for PRO-C6 (*P* = 0.75), PRO-C6% change (*P* =  0.60), C6M (*P* =  0.53) and for C6M percent change (*P* = 0.77). Significant average contrast across all timepoints between treatment and non-treatment for PRO-C6 and C6M is shown as * (*P* < 0.05)

Serum PRO-C6 levels were significantly higher (*P* = 0.031) in the untreated group in comparison with the treated group over 12 months with an average difference across all timepoints of 13% (95% CI 1–26) (Fig. 2A). However, there was no difference when analyzing the percent change from baseline (Fig. 2C). Serum C6M levels were significantly higher (*P* = 0.017) in the untreated group in comparison with treated group over 12 months with an average difference across all timepoints of 21% (95% CI 3–41) (Fig. 2B). Furthermore, the change from baseline in C6M in untreated patients was significantly increased (*P* = 0.043) from baseline to 12 months with an average difference across all timepoints of 24% (95% CI -0.07-47.2) compared to patients on treatment (Fig. 2D). Moreover, in subgroup analyses biomarker levels were compared within progressive patients between treated and untreated patients. PRO-C6 and C6M levels were numerically higher in untreated patients with a progressive disease compared to treated patients with a progressive disease, however, a statistically significant difference (*P* = 0.017) was only reached with C6M over 12 months with an average difference across all timepoints of 34% (95% CI 5–71) (Additional file 1: Fig. S3 A-B). Furthermore, in subgroup analyses only including stable patients, PRO-C6 levels were significantly higher (*P* = 0.038) in untreated patients over 12 months with an average difference across all timepoints of 16% (95% CI 1–34) compared to treated patients while C6M did not show any differences (Additional file 1: Fig. S3 C-D).

**Fig. 3:**
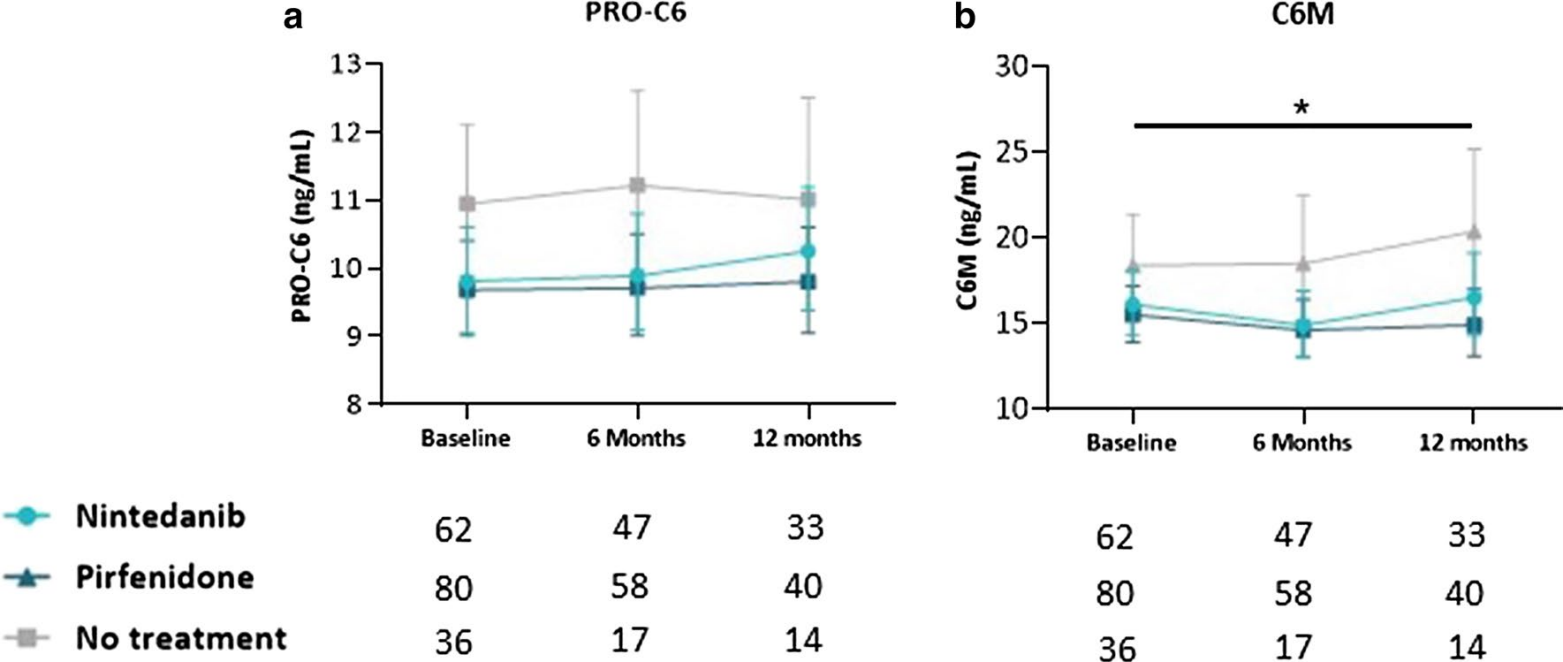
Longitudinal levels of type VI collagen biomarkers in nintedanib, pirfenidone and untreated IPF patients. Serum levels of PRO-C6 (**A**) and C6M (**B**) are shown at baseline, six months and 12 months for pirfenidone (dark turquoise), nintedanib (turquoise) and non-treatment (grey) patients with IPF. Data are presented as geometric mean with 95% CI (error bars) adjusted for age and sex. The number of evaluable samples available for analysis at each time point is provided below the graphs. The interaction between timepoint and treatment was not significant forPRO-C6 (*P* = 0.87) and for C6M (*P* = 0.70). A significant average contrast across all timepoints between pirfenidone and non-treatment for C6M is shown as * (*P* < 0.05)

No differences in PRO-C6 or C6M levels were observed over 12 months between patients receiving pirfenidone or nintedanib (Fig. 3A, B). Serum PRO-C6 and C6M levels were numerically lower over 12 months in patients receiving pirfenidone or nintedanib compared to patients without antifibrotic treatment, but statistical significance (*P* =  0.036) was only observed for C6M with an average difference across all timepoints of 23.3% (95% CI 1–50) between the pirfenidone and no treatment group (Fig. 3A, B).

## Discussion

Remodeling of the ECM is a central mechanism in the progression of IPF, and remodeling of type VI collagen has been suggested to be associated with disease progression [20, 22]. In the current study, we investigated biomarkers reflecting type VI collagen formation and endotrophin (PRO-C6) as well as type VI collagen degradation (C6M) in a real-world cohort of incident IPF patients enrolled prior to treatment initiation. We showed that type VI collagen formation and to some extent degradation were elevated in patients with progressive IPF compared with stable IPF over 12 months. These results are in line with previous findings from the PROFILE cohort [18, 20]. The present analyses were performed in a real-world cohort, and a less strict definition of progression was used in order to also include patients with marginal physiological progression of disease. Despite using this broad definition of progression, the biomarkers were still elevated in patients with progressive disease similar to previous findings, where a more conservative definition of progression was used. Our study confirms that elevated type VI collagen formation and degradation are associated with progression of IPF, also in early, less advanced disease. In addition, marginal declines in FVC and DLCO have been proven to predict mortality in IPF patients [27, 28]. Also, it has been estimated that the minimal clinically important difference in IPF patients is between 2 and 6% change in % predicted FVC [29].

We compared longitudinal biomarker levels of PRO-C6 and C6M between untreated patients and the combined treatment group of patients on antifibrotic treatment; the latter had lower values of PRO-C6 and C6M over 12 months compared with the patients without treatment. Furthermore, when we analysed only the progressive patients, the untreated ones had higher levels of C6M; the analyses with only stable patients showed that the untreated group had higher PRO-C6 levels, yet these analyses were limited to a small number of patients of the untreated group. The reason for this difference between the groups is presently unknown and may represent intrinsic differences between the two groups that are not explained by our data or merely due to chance. Patients without treatment were either patients with very mild disease choosing not to start treatment or patients with very advanced disease. Our data could indicate that the majority of patients in the non-treated group have more active disease with higher biomarker levels and a shorter 6MWT distance, yet this is not reflected in the commonly applied physiological variables FVC and DLCO which declined similarly in the treated and untreated groups. Other real-life studies have also reported similar findings [30]. Furthermore, we did not find any differences in the biomarker levels between nintedanib and pirfenidone treated patients, and the PRO-C6 and C6M levels seemed to be stable over 12 months in both groups of patients. This could be due to the relatively less advanced disease of patients in this cohort. It would be interesting to examine PRO-C6 and C6M in a treatment cohort with more advanced disease.

Finally, when we looked at the change from baseline to follow-up in biomarker levels, C6M increased in patients without treatment and remained unchanged in the treated patients. This could indicate that nintedanib and pirfenidone potentially affects the degradation of type VI collagen. Moreover, C6M measures a neoepitope generated by MMP-2 and -9 degradation of type VI collagen [21] and indirectly it may indicate that nintedanib and pirfenidone could slow down the MMP-2 and -9 activity. In contrast to our finding, it was shown in a small proof of concept study in IPF patients that treatment with omipalisib reduced the levels of PRO-C6 but not C6M [31]. However, this potential antifibrotic treatment has a different mode of action and the study was done in a short time frame compared to PFBIO and this may explain some of the differences.

An important strength of our study is the prospective longitudinal design, in which the collection of blood samples is standardized. Also, we investigate biomarkers in a real-world cohort with less advanced disease comparable to many IPF trial cohorts. On the other hand, a weakness of the current study was that the analyses were only performed in a single cohort with no validation cohort. Furthermore, only few patients progressed when using 10% decline over 12 months, and we, therefore, used a 5% decline to define a progressive phenotype. However, both PRO-C6 and C6M have previously been linked to progression when using the definition of 10% decline in FVC, and the current study confirms that PRO-C6 and C6M are associated with IPF progression and are relevant biomarkers to study further in IPF. The fact that baseline biomarker levels were elevated in untreated patients compared to treated patients was a limitation, and this could affect the interpretation of treatment effects on the biomarkers. We have tried to compensate for this by adjusting for baseline biomarker levels.

## Conclusions

In conclusion, this study demonstrated that type VI collagen formation is related to the progression of IPF in a real-world cohort. This could provide valuable information for clinicians to identify patients at high risk of disease progression. Furthermore, this study indicates that antifibrotic treatment might normalize type VI collagen degradation. Yet, further studies preferably with larger sample size and longer disease durations are required to validate these findings.

## Supplementary Information

**Additional file 1: Table S1.** Baseline characteristics. **Fig. S1.** Flow diagram. **Fig. S2.** Longitudinal biomarker levels are elevated in progressive IPF patients (without death). **Fig. S3.** Longitudinal levels of type VI collagen biomarkers in treated and untreated IPF patients. **Fig. S4.** Risk of disease progression at 12 months for IPF patients. **Fig. S5.** Risk of disease progression at 12 months for IPF patients with change in biomarker levels.

## Data Availability

The datasets generated and/or analysed during the current study are not publicly available due to restrictions by the Danish data protection laws but are available from the corresponding author on reasonable request.

## Abbreviations

BMI: Body mass index
CI: Confidence interval
C6M: Collage type VI degraded by matrix metalloprotease
DLco: Diffusion capacity for carbon monoxide
ECM: Extra cellular matrix
FVC: Forced vital capacity
GAP index: Gender, age, and physiology index
ILD: Interstitial lung disease
IPF: Idiopathic pulmonary fibrosis
MMP: Matrix metalloprotease
PFBIO: Pulmonary Fibrosis Biomarker
PRO-C6: Collagen synthesis neoepitopes for collagen-type VI
6MWT: 6-minute walk test
