# Supplementary material for: Longitudinal serological assessment of type VI collagen turnover is related to progression in a real-world cohort of idiopathic pulmonary fibrosis
Source: BMC Pulm Med. 2021 Nov 23;21:382. doi: 10.1186/s12890-021-01684-3 (PMC8609852; doi:10.1186/s12890-021-01684-3)
Supplement: Supplementary file 1 — Additional file 1: Table S1. Baseline characteristics. Fig. S1. Flow diagram. Fig. S2. Longitudinal biomarker levels are elevated in progressive IPF patients (without death). Fig. S3. Longitudinal levels of type VI collagen biomarkers in treated and untreated IPF patients. Fig. S4. Risk of disease progression at 12 months for IPF patients. Fig. S5. Risk of disease progression at 12 months for IPF patients with change in biomarker levels. [file 12890_2021_1684_MOESM1_ESM.pdf]

Additional file 1: Table S1

Parameters	All patients n=178	Progressive n=84	Stable n=94	p-value
**Age, mean (SD)**	73.80 (7.53)	73.31 (7.55)	74.23 (7.53)	0.415
**Men, n (%)**	136 (76.4%)	69 (82.1%)	67 (71.3%)	0.126
**BMI, mean (SD)**	27.31 (4.51)	27.19 (4.53)	27.41 (4.51)	0.744
**FVC (L), mean (SD)**	3.04 (0.86)	3.13 (0.88)	2.96 (0.85)	0.17
**FVC (% pred), mean (SD)**	89.51 (19.52)	88.74 (20.51)	90.20 (18.67)	0.448
**DLCO (% pred), mean (SD)**	52.85 (13.25)	52.73 (13.31)	52.96 (13.27)	0.645
**Change in FVC% pred. from baseline to 12 months, mean (SD)**	0.13 (10.16)	-6.98 (7.61)	5.60 (8.34)	<0.0001
**Change in DLCO% pred. from baseline to 12 months, mean (SD)**	-5.03 (7.98)	-11.25 (6.90)	-0.26 (4.85)	<0.0001
**6MWT (meters), mean (SD)**	442.61 (105.58)	441.63 (104.92)	443.50 (106.74)	0.394
**Smoking status, n (%)**				0.262
**Never**	46 (25.8%)	17 (20.2%)	29 (30.9%)	
**Active**	11 (6.2%)	6 (7.1%)	5 (5.3%)	
**Former**	121 (68.0%)	61 (72.6%)	60 (63.8%)	
**GAP index, n (%)**				0.117
**I**	88 (49.7%)	40 (47.6%)	48 (51.6%)	
**II**	82 (46.3%)	38 (45.2%)	44 (47.3%)	
**III**	7 (4.0%)	6 (7.1%)	1 (1.1%)	

Additional file 1: Table S1: Baseline characteristics

SD standard deviation, BMI Body Mass Index, FVC forced vital capacity, DLCO diffusion capacity for carbon monoxide, 6MWT six-minute walk test, GAP index Gender-Age-Physiology index, Disease progression was defined as ≥ 5% decline in FVC and/or ≥ 10% decline in DLCO or all-cause mortality within 12 months.

Additional file 1: Fig. S1

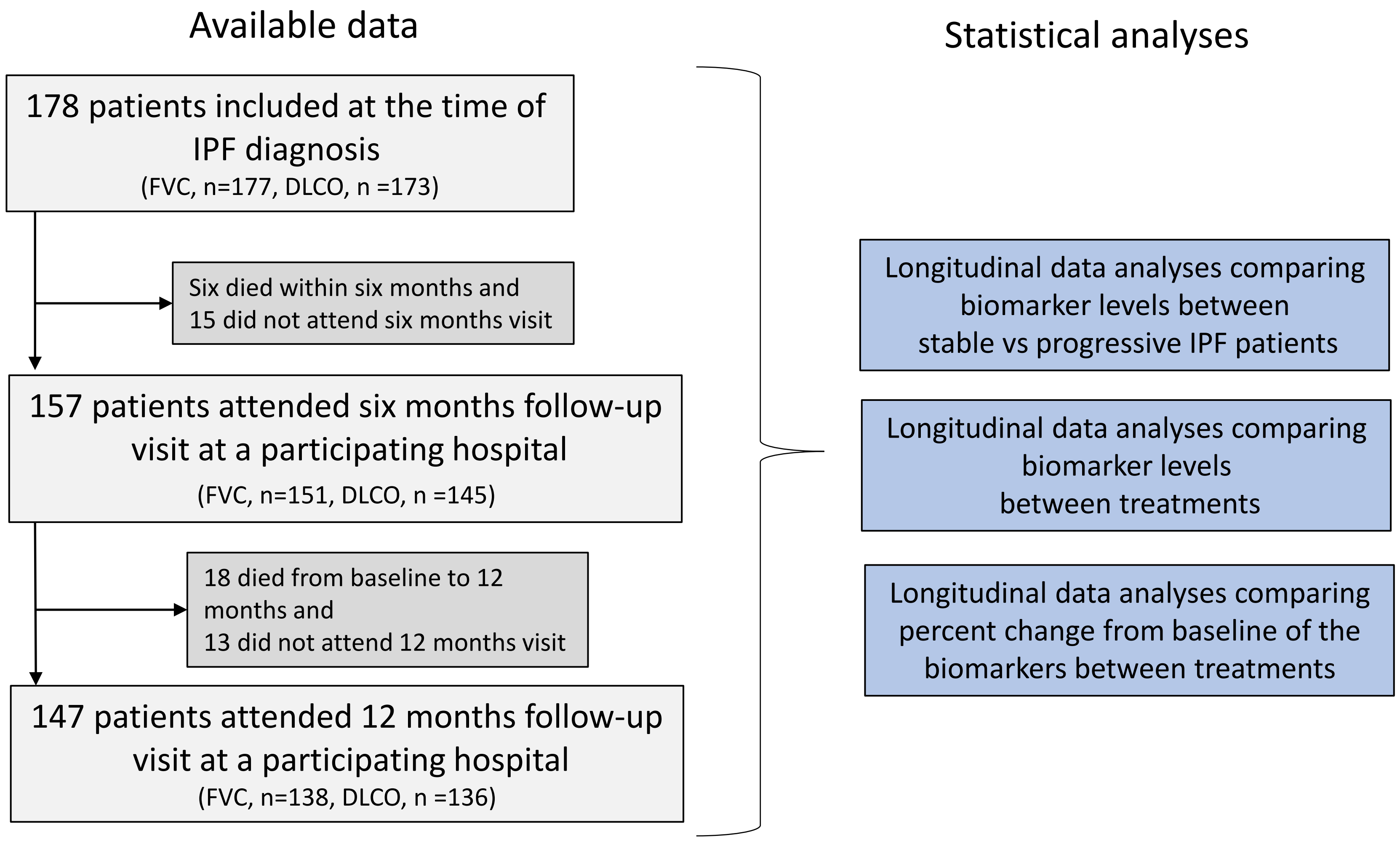

Additional file 1: Fig. S1 : Flow diagram
Follow-up of patients included in the presented study.

Additional file 1: Fig. S2

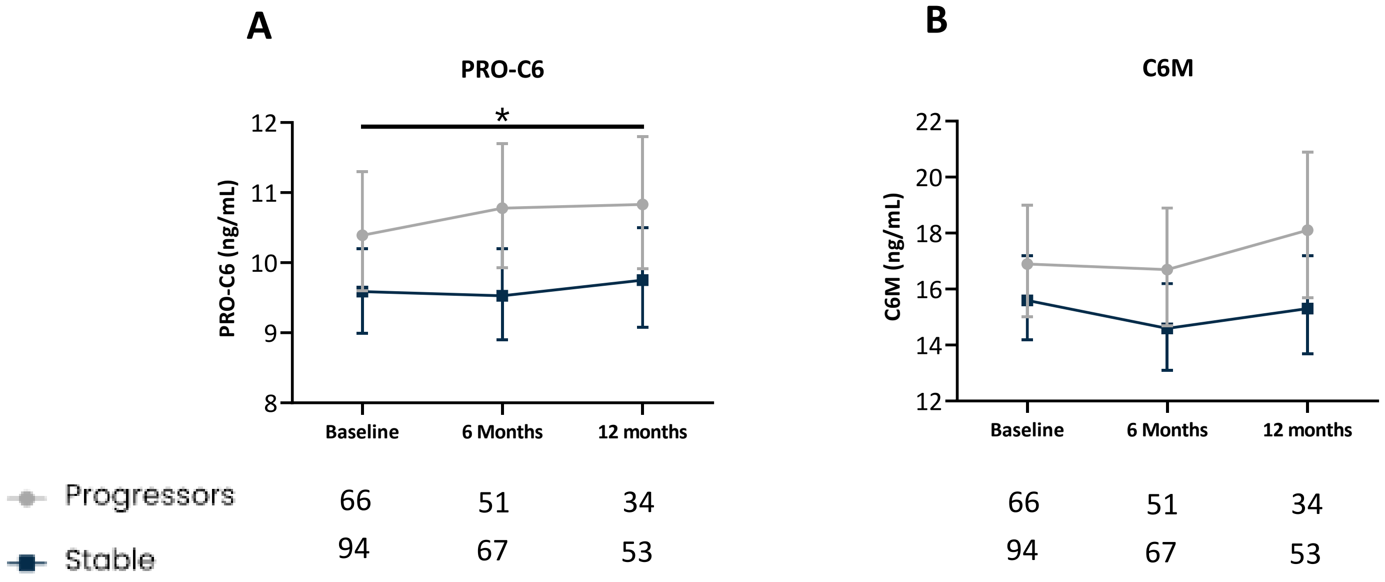

Additional file 1: Fig. S2: Longitudinal biomarker levels are elevated in progressive IPF patients (without death).
*Serum levels of PRO-C6 (A), and C6M (B) are shown at baseline, six months and 12 months for stable (dark blue) and progressive (grey) patients with IPF. Disease progression was defined as ≥ 5% decline in FVC and/or ≥ 10% decline in DLco within 12 months. Data are presented as geometric mean with 95% CI (error bars) adjusted for age and sex. The number of evaluable samples available for analysis at each time point is provided below the graphs. The interaction between timepoint and progression status was not significant for PRO-C6 (P=0.43) and for C6M (P=0.51). A significant average contrast across all timepoints between stable and progressors for PRO-C6 is shown as * (P<0.05).*

Additional file 1: Fig. S3

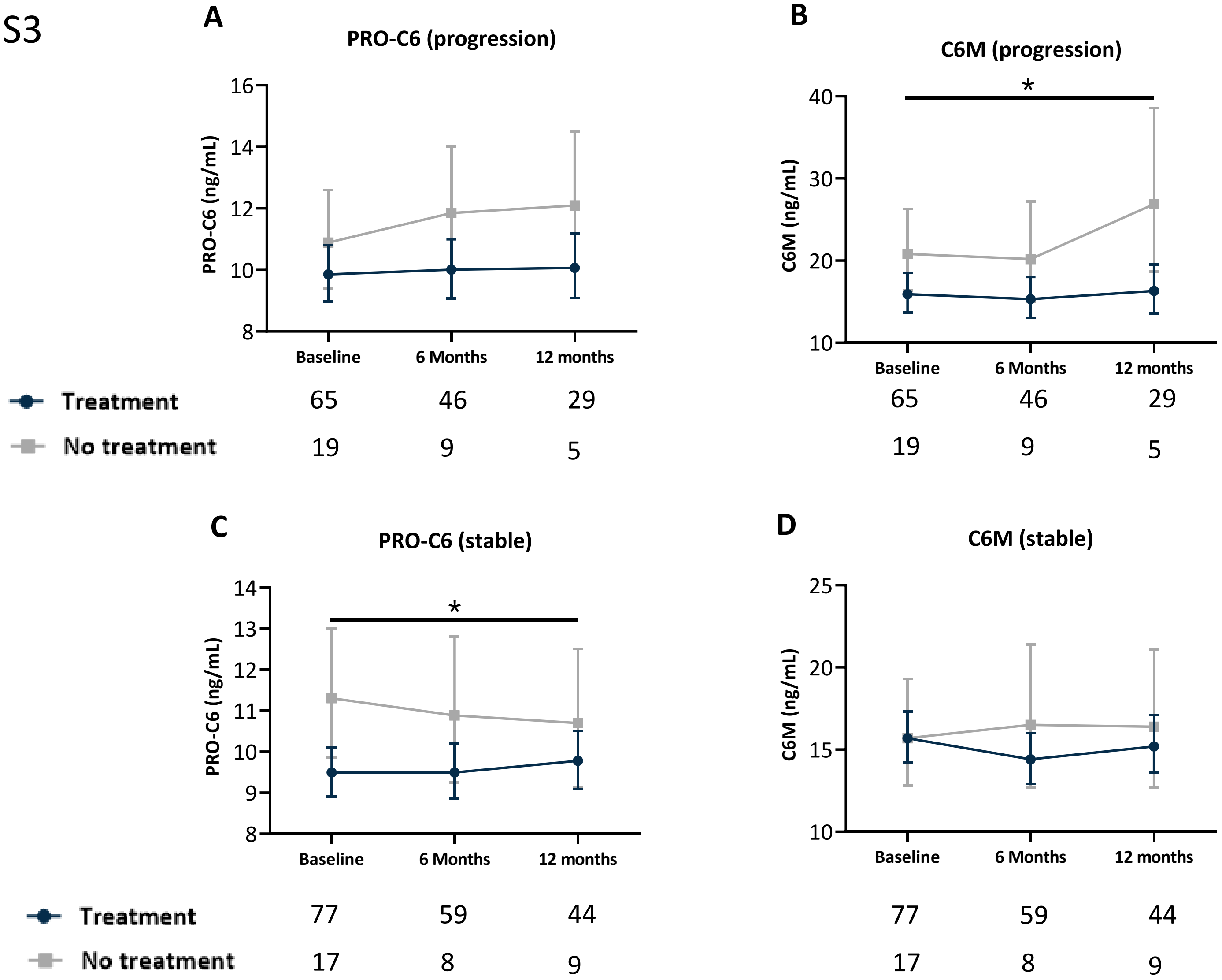

Additional file 1: Fig. S3: Longitudinal levels of type VI collagen biomarkers in treated and untreated IPF patients. *Levels of PRO-C6 (A) and C6M (B) in patients with a progressive disease and levels of PRO-C6 (C) and C6M (D) in patients with a stable disease at baseline, six months and 12 months for treated (nintedanib/pirfenidone) (dark blue) and for non-treated (grey). Data are presented as mean and 95% CI (error bars) adjusted for age and sex. The number of evaluable samples available for analysis at each time point is provided in the graph. The interaction between timepoint and treatment was not significant for PRO-C6 progression (P=0.37), PRO-C6 stable (P=0.46), C6M progression (P=0.41) and for C6M stable (P=0.59). Significant average contrast across all timepoints between treatment and non-treatment for PRO-C6 and C6M are shown as* * *(P<0.05).*

Additional file 1: Fig. S4

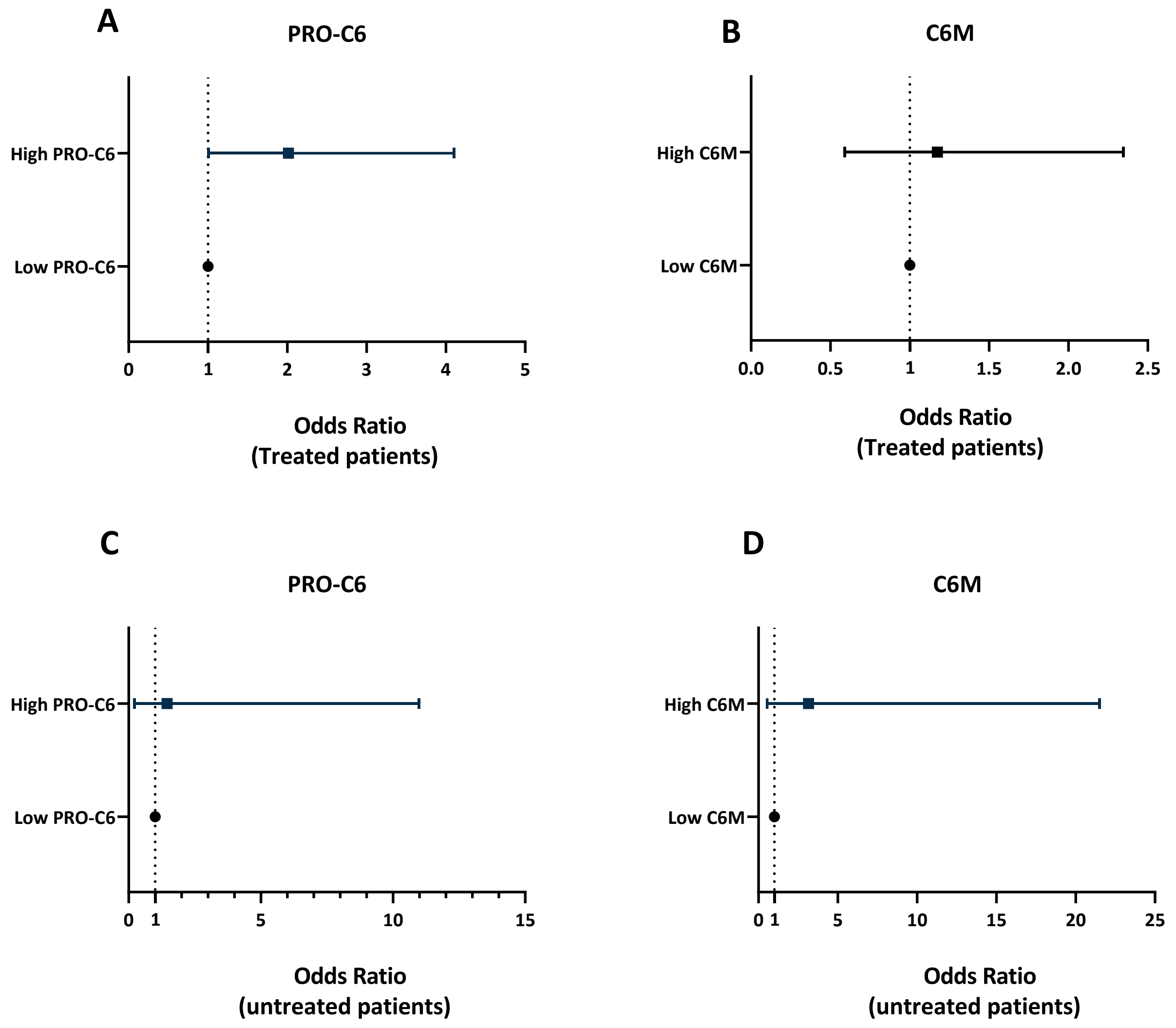

Additional file 1: Fig. S4: Risk of disease progression at 12 months for IPF patients.

Odds ratio from IPF patients divided into high or low baseline biomarker for treated or untreated patients of PRO-C6 (A, C) and C6M (B, D) are shown for the high biomarker compared to the low biomarker. Disease progression was defined as ≥ 5% decline in FVC and/or ≥ 10% decline in DLco or all-cause mortality at 12 months. Data are presented as mean and 95% CI (error bars) adjusted for age, sex, and baseline levels of FVC and DLco.

Additional file 1: Fig. S5

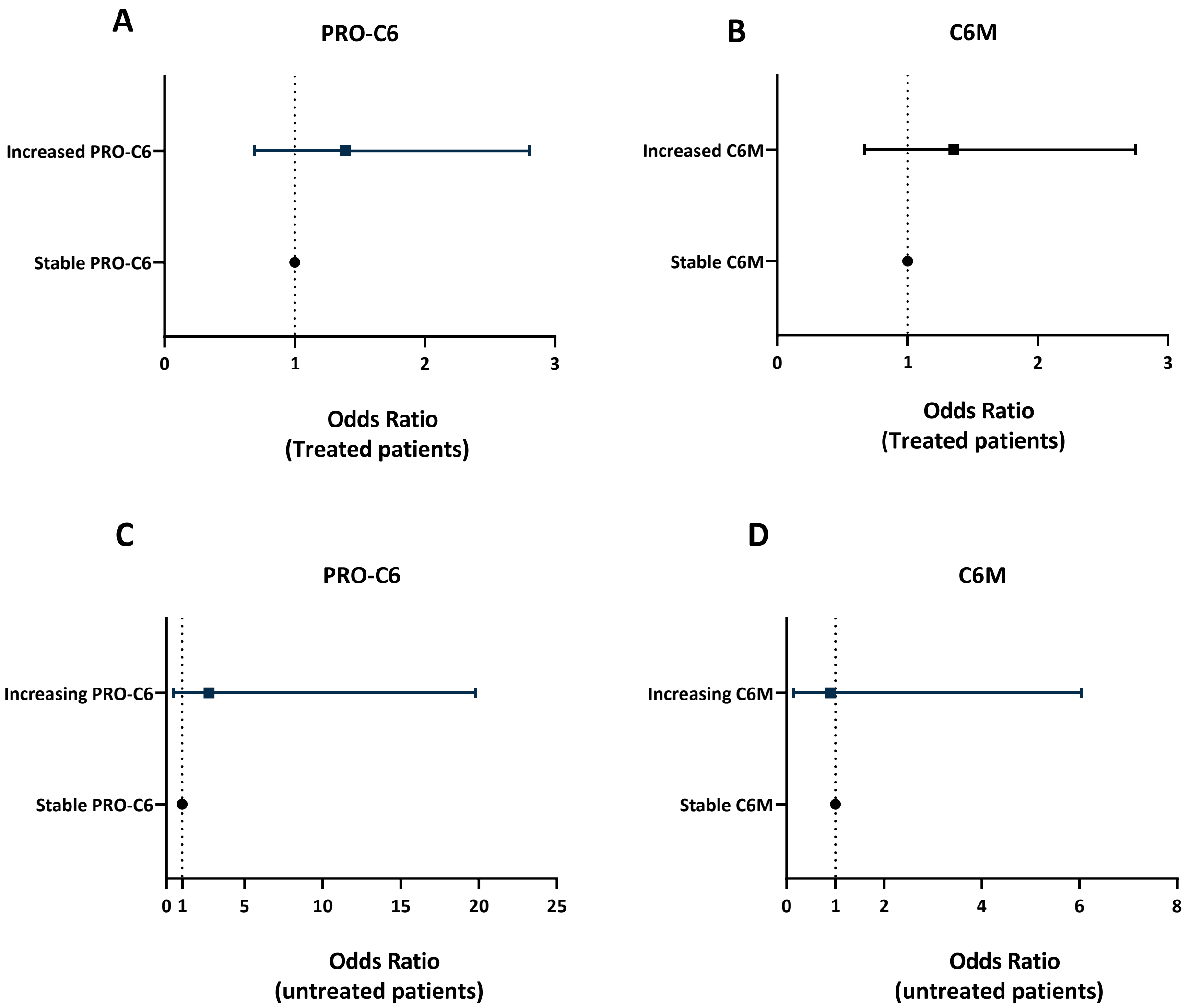

Additional file 1: Fig. S5: Risk of disease progression at 12 months for IPF patients with change in biomarker levels.
Odds ratio from IPF patients divided into stable or increasing biomarker levels during 12 months for treated or untreated patients of PRO-C6 (A, C) and C6M (B, D) are shown for the increasing biomarker compared to the stable biomarker. Disease progression was defined as ≥ 5% decline in FVC and/or ≥ 10% decline in DLco or all-cause mortality at 12 months. Data are presented as mean and 95% CI (error bars) adjusted for age, sex, and baseline levels of FVC and DLco.
